# β-blockers Reverse Agonist-Induced β_2_-AR Downregulation Regardless of Their Signaling Profile

**DOI:** 10.3390/ijms21020512

**Published:** 2020-01-14

**Authors:** Sonia Maccari, Vanessa Vezzi, Federica Barbagallo, Tonino Stati, Barbara Ascione, Maria Cristina Grò, Liviana Catalano, Giuseppe Marano, Paola Matarrese, Caterina Ambrosio, Paola Molinari

**Affiliations:** 1 Center for Gender-Specific Medicine, National Institute of Health, 00161 Rome, Italy; 2 National Center for Drug Research and Evaluation, National Institute of Health, 00161 Rome, Italy; 3 Department of Experimental Medicine, Sapienza University, 00161 Rome, Italy; 4 National Blood Center, 00161 Rome, Italy

**Keywords:** pharmacology, cell surface receptor density, β-blockers, cultured cells, lymphocytes

## Abstract

Altered β-adrenergic receptor (β-AR) density has been reported in cells, animals, and humans receiving β-blocker treatment. In some cases, β-AR density is upregulated, but in others, it is unaffected or even reduced. Collectively, these results would imply that changes in β-AR density and β-blockade are not related. However, it has still not been clarified whether the effects of β-blockers on receptor density are related to their ability to activate different β-AR signaling pathways. To this aim, five clinically relevant β-blockers endowed with inverse, partial or biased agonism at the β_2_-AR were evaluated for their effects on β_2_-AR density in both human embryonic kidney 293 (HEK293) cells expressing exogenous FLAG-tagged human β_2_-ARs and human lymphocytes expressing endogenous β_2_-ARs. Cell surface β_2_-AR density was measured by enzyme-linked immunosorbent assay (ELISA) and flow cytometry. Treatment with propranolol, carvedilol, pindolol, sotalol, or timolol did not induce any significant change in surface β_2_-AR density in both HEK293 cells and human lymphocytes. On the contrary, treatment with the β-AR agonist isoproterenol reduced the number of cell surface β_2_-ARs in the tested cell types without affecting β_2_-AR-mRNA levels. Isoproterenol-induced effects on receptor density were completely antagonized by β-blocker treatment. In conclusion, the agonistic activity of β-blockers does not exert an important effect on short-term regulation of β_2_-AR density.

## 1. Introduction

Catecholamines, acting through α- and β-adrenergic receptors, regulate many physiological functions, such as force and frequency of cardiac contraction, vascular and bronchial tone, and metabolism, and are an essential component of the body’s stress response. The tissue responses to catecholamines depend on their relative affinities for receptor adrenergic subtypes, their concentration at the site of the receptor as well as the adrenergic receptor density on target cells. Drugs, such as β-blockers, and clinical disorders, such as heart failure, can alter the number of β-adrenergic receptors (β-AR). Previous studies performed on both animals and humans have reported dissimilar effects of β-blocker therapy on β-AR density and sensitivity. Chronic treatment with the β-AR antagonist propranolol significantly increased the number of β-ARs in the heart, lung, and lymphocytes [1,2,3,4]. On the contrary, the administration of pindolol, another β_1_ - and β_2_ -AR antagonist, resulted in a decrease in the lymphocyte β-AR density [2,3]. Furthermore, treatment with carvedilol, a third-generation β-blocker, was not able to affect myocardial β-AR density in patients with heart failure [5]. Together, these results would imply that changes in receptor density and β-blocking activity of β-blockers are not related. However, it remains to be ascertained whether β-blocker-induced changes in receptor density are related to other pharmacological properties of β-blockers.

Recent findings in the molecular biology of β-ARs show that these receptors can interact with at least two different transduction proteins in the cell membrane: G proteins and β-arrestins. Endogenous agonists of G protein-coupled receptors (GPCRs) are usually equally efficient in promoting G protein-mediated signaling and the interaction of the receptor with arrestins, which promote rapid receptor internalization and signal quenching. However, GPCR ligands, such as β-blockers, can show unequal or divergent molecular efficacies for such interactions. This phenomenon, often called biased agonism or functional ligand selectivity [6], indicates that GPCR ligands do not have a unique profile of efficacy, as previously thought, but can act as agonists or antagonists on distinct transducers, and thus generate a complex pattern of signaling and biological effects. In the present study, we determined whether there is a correlation between the signaling profile of β-blockers and their ability to perturb cell surface β-AR density.

β-ARs (β_1_-ARs, β_2_-ARs, and β_3_-ARs) belong to the GPCR superfamily and activate adenylyl cyclase following the binding with catecholamines. β-AR signaling is terminated by phosphorylation of the intracellular domains of the receptor by the family of G protein-coupled receptor kinases. Although both β_1_- and β_2_-AR couple primarily to Gαs and subsequent cyclic adenosine monophosphate (cAMP)-related pathways, they are regulated differently in response to persistent agonist stimulation. In general, the studies in cultured cells indicate that β_1_-AR is more resistant to agonist-mediated downregulation than β_2_-AR [7,8,9]. Since β_2_-ARs are distributed extensively throughout the body, wherein they mediate the response to sympathetic discharges in many organs and tissues, including immune and central nervous systems [10,11,12], there is considerable interest in their regulation. At present, an in vitro analysis of the effects of β-blockers with different signaling profiles on β_2_-AR density is lacking.

To this aim, five clinically relevant β-blockers endowed with inverse, partial, or biased agonism at the β_2_-AR, according to Wisler and colleagues [13], were tested in both human embryonic kidney 293 (HEK293) cells transfected with FLAG-tagged human β_2_-ARs and human lymphocytes that express endogenous β_2_-ARs. Cell surface β_2_-AR density was measured by enzyme-linked immunosorbent assay (ELISA) and flow cytometry using specific antibodies. We found that propranolol, carvedilol, pindolol, sotalol, or timolol do not affect β_2_-AR density on their own but reverse agonist-induced β_2_-AR downregulation.

## 2. Results

### 2.1. β-Blockers Do Not Affect Receptor Density in Simple Cell Systems

**Figure 1:**
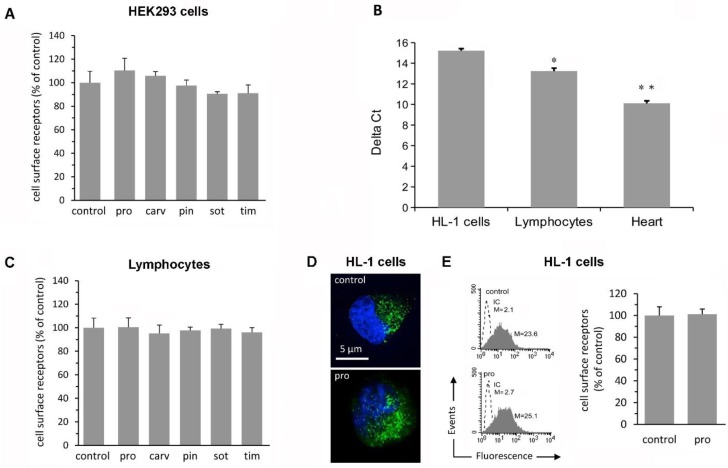
β-blockers do not affect cell surface β-adrenergic receptor (β_2_-AR) density. (**A**) Bar graph showing the effects of β-blockers on cell-surface β_2_-AR density. HEK293 cells stably expressing the N-terminal-FLAG-β_2_-AR were treated at 37 °C for 3 h with β-AR antagonists [propanolol (pro), carvedilol (car), pindolol (pin), sotalolol (sot), timolol (tim)] at the concentration of 1 μM. Treatment with β-AR antagonists did not affect surface β_2_-AR density. Data are means ± SEM from three independent experiments. (**B**) β_2_-AR gene expression in HL-1 cells and peripheral blood lymphocytes was analyzed by real-time polymerase chain reaction (qPCR). β_2_-AR gene was expressed in both HL-1 cells and peripheral blood lymphocytes. In these cells, β_2_-AR gene expression was markedly smaller than in the heart. Delta Ct=Ct (gene of interest) – Ct (reference gene); Ct=cycle threshold. *p < 0.05 vs. HL-1 cells; **p < 0.05 vs. HL-1 cells and lymphocytes. (**C**) Bar graph showing the effects of β-blockers on cell-surface β_2_-AR density of human lymphocytes. Data are means ± SEM from three independent experiments. (**D**) Intensified video microscopy analysis after cell staining with anti-β_2_-AR antibody (green) and counterstained with Hoechst (blue) in HL-1 cells. (**E**) Cytometric analysis of β_2_-AR density in HL-1 cells. (left panel) A representative experiment of quantitative evaluation of β_2_-AR density, after specific immunostaining, using flow cytometric analysis. M stands for median fluorescence intensity (MFI). Dashed line: cells plus isotypic immunoglobulines followed by secondary antibody. (right panel) Bar graph showing cytofluorimetric results reported as mean ± SEM, from three independent experiments and expressed as a percentage of the value obtained in untreated cells (control).

To determinate the effects of β-blockers on β_2_-AR density, we used the HEK293 cell line, stably expressing the N-terminus-FLAG-tagged β_2_-AR at a density of 15 pmol/mg of membrane protein as previously reported [14]. A panel of five β-blockers (propanolol, carvedilol, pindolol, sotalol, and timolol) with different efficacy profiles for both cAMP generation and extracellular signal-regulated kinases (ERK) activation, according to the classification suggested by Wisler et al. [13], was used to evaluate β-blocker ability in changing β_2_-AR density. Specifically, we tested pindolol, a partial agonist, sotalol, and timolol, two inverse agonists, carvedilol and propranolol, two inverse agonists that also induce ERK activation, i.e., biased agonists [13]. As shown in Figure 1A, no tested β-blocker altered β_2_-AR density in HEK293 cells. Furthermore, although propranolol tended to increase β_2_-AR density no statistical difference was observed among β-blockers (Figure 1A)

Given that the regulation of β-ARs expressed by endogenous genes may be more relevant to in vivo receptor regulation, we next evaluated the effects of β-blockers on β_2_-AR density of endogenous β_2_-ARs expressed in human lymphocytes. These cells have β_2_-ARs [15], whose expression level is much lower than in the heart (Figure 1B). Again, no tested β-blocker at the concentration of 1 μM induced any significant change in surface β_2_-AR density in lymphocytes (Figure 1C).

Since most studies on the effects of β-blockers on receptor density were performed using the β-blocker propranolol [1,2,3,4,16,17], we also evaluated the effects of propranolol on the density of endogenous β_2_-ARs expressed in HL-1 atrial cardiomyocytes derived from mouse AT-1 cells. These cells are currently the only cardiomyocyte cell line available that can be serially passaged while maintaining a differentiated cardiac phenotype. Similarly to lymphocytes, HL-1 cells have β_2_-ARs [18], but their expression level is much lower than in the heart (Figure 1B). Propranolol treatment at the concentration of 1 μM did not induce any significant change in surface β_2_-AR density in HL-1 cells as determined by static (Figure 1D) and flow cytometry (Figure 1E). Collectively, these results indicate that treatment with β-blockers does not affect short-term regulation of β_2_-AR density in three different cell types.

### 2.2. β-Blockers Restore β_2_-AR Density after Agonist-Induced Receptor Downregulation

**Figure 2:**
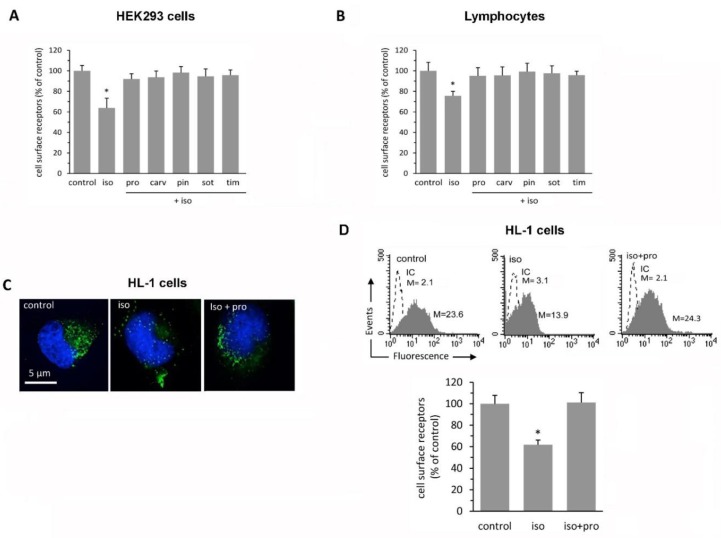
β-blockers restore cell surface β_2_-AR density after agonist-induced receptor downregulation. (**A**) Bar graph showing the effects of the β_2_-AR agonist isoproterenol on cell-surface β_2_-AR density. HEK293 cells stably expressing the N-terminal-FLAG-β_2_-AR were treated at 37 °C for 3 h with isoproterenol (iso) at the concentration of 1 μM. Isoproterenol caused receptor downregulation. This effect was completely antagonized upon adding β-AR antagonists. Data are means ± SEM from three independent experiments. Abbreviations as in Figure 1. * *p* < 0.05 vs. all other groups. (**B**) Bar graph showing the effects of the β_2_-AR agonist isoproterenol on the cell-surface β_2_-AR density of human lymphocytes. Isoproterenol caused receptor downregulation, but this effect was completely antagonized upon adding β-AR antagonists. Data are means ± SEM from three independent experiments. Abbreviations as in Figure 1. * *p* < 0.05 vs. all other groups. (**C**) Intensified video microscopy analysis after cell staining with anti-β_2_-AR antibody (green) and counterstained with Hoechst (blue) in HL-1 cells. Isoproterenol reduces β_2_-AR density, but the addition of propranolol to the culture medium reverses isoproterenol-mediated β_2_-AR downregulation. (**D**) Cytometric analysis of β_2_-AR density in HL-1 cells. Again, isoproterenol reduces β_2_-AR density, but propranolol antagonizes it. (upper panel) A representative experiment of quantitative evaluation of β_2_-AR density, after specific immunostaining, using flow cytometric analysis. M stands for median fluorescence intensity (MFI). Dashed line: cells plus isotypic immunoglobulines followed by secondary antibody. (lower panel) Bar graph showing cytofluorimetric results reported as mean ± SEM from three independent experiments and expressed as a percentage of the value obtained in untreated cells (control). * *p* < 0.05 vs. all other groups.

Treatment with catecholamines causes β_2_-AR downregulation. To obtain the maximal receptor downregulation, HEK 293 cells stably expressing the N-terminus-FLAG-tagged β_2_-AR were incubated in the presence of 1 μM isoproterenol, a synthetic β-AR agonist catecholamine, at 37 °C per 3 h according to a previous study [14]. Approximately 40% of β_2_-ARs were downregulated in response to isoproterenol stimulation, but, upon adding of β-blockers, internalized receptors completely recycled back to the plasma membrane (Figure 2A). All the tested β-blockers showed equal efficacy in restoring receptor density regardless of their ancillary pharmacological properties.

We next evaluated the effects of the β-AR agonist isoproterenol and β-AR antagonists on β_2_-AR density in human lymphocytes. Incubation of these cells with isoproterenol (1 μM) for 3 h reduced β_2_-AR density compared to cells incubated with control medium (Figure 2B). To address the question of whether β-blockers restore β_2_-AR density in isoproterenol-treated cells, lymphocytes were incubated with isoproterenol for 3 h before adding β-blockers (1 μM) for another 30 min. Coincubation of the cells with isoproterenol and β-blockers (propranolol, carvedilol, pindolol, sotalol, or timolol) completely reversed isoproterenol-mediated β_2_-AR downregulation restoring surface receptor density (Figure 2B).

We also evaluated the effects of isoproterenol on β_2_-AR density in HL-1 cardiac cells. We found that treatment with the β-AR agonist isoproterenol reduced the number of cell surface β_2_-ARs in HL-1 cells as determined by static (Figure 2C) and flow cytometry (Figure 2D). The isoproterenol-induced effects on receptor density were completely antagonized by propranolol treatment (Figure 2C,D). Collectively, these results suggest that β-blockers could exert a class effect in reversing isoproterenol-induced β_2_-AR downregulation in these simple cellular models.

### 2.3. Isoproterenol Did Not Affect β_2_-AR Gene Expression

**Figure 3:**
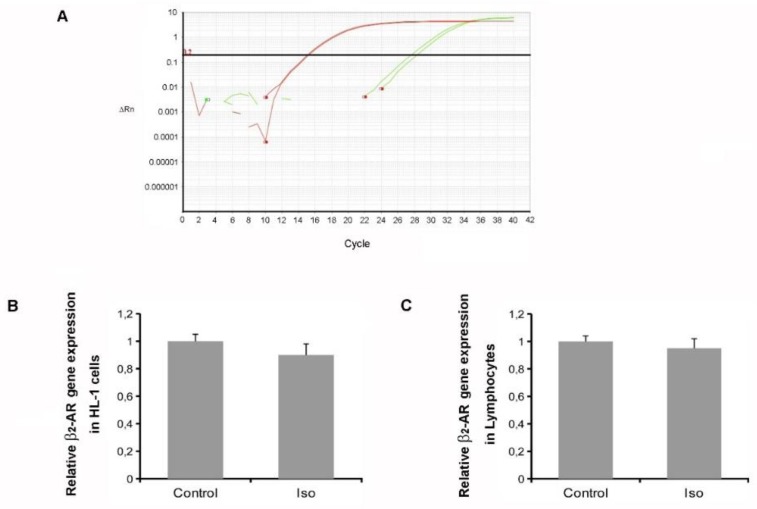
Isoproterenol does not affect β_2_-AR mRNA levels in both HL-1 cells and lymphocytes. (**A**) Representative amplification plot for TaqMan qPCR using the β_2_-AR assay probe in human lymphocytes (red line, reference gene; green line, β_2_-AR gene). (**B**) β_2_-AR gene expression in HL-1 cells treated with isoproterenol (iso) (1 μM) for 3 h. For each group, summary data of three independent experiments run in duplicate are shown. (**C**) β_2_-AR gene expression in lymphocytes treated with isoproterenol (1 μM) for 3 h. For each group, summary data of three independent experiments run in duplicate are shown.

We performed TaqMan real-time polymerase chain reaction (qPCR) to assess the mRNA levels of β_2_-ARs in both HL-1 cardiac cells and lymphocytes (Figure 3A). In both cell types, treatment with isoproterenol (1 μM) for 3 h was not able to change β_2_-AR gene expression (Figure 3B,C). Together, these results suggest that isoproterenol-mediated β_2_-AR downregulation is not attributable to changes in receptor *transcriptional regulation.*

## 3. Discussion

Regulation of β-AR plasma membrane density is an important process in tuning β-AR response to catecholamines. β-AR antagonists inhibit catecholamine-induced β-AR stimulation and are commonly used to treat a variety of clinical conditions ranging from heart failure to capillary hemangioma. Chronic treatment of humans, animals, or cells with β-blockers may increase, reduce, or even leave the number of β-ARs unchanged depending on the drug used [1,2,3,4,5]. Although chronic adaptations of cardiovascular and sympathetic nervous systems resulting from long-term β-AR blockade can contribute to altered receptor density in animals or humans receiving chronic β-blocker treatment, they do not explain all facets of the problem. Since recent findings in the molecular biology of β-ARs show that β-ARs can interact with G proteins and β-arrestins and that β-blockers can show unequal or divergent molecular efficacies for such interactions, we tested the hypothesis that there is a relation between signaling profile of β-blockers and their ability to perturb cell surface β-AR density. To this aim, five clinically relevant β-blockers endowed with inverse, partial, or biased agonism at the β_2_-AR were evaluated for their effects on β_2_-AR density in both cells expressing exogenous FLAG-β_2_-ARs and cells expressing endogenous β_2_-ARs. Changes in receptor density were evaluated by immunological techniques, such as ELISA and flow cytometry. Using these techniques and these simple cell systems, we found that this subset of β-blockers does not affect short-term regulation of β_2_-AR density but is able to reverse agonist-induced β_2_-AR downregulation.

The results of the present study are in line with those of previous studies that observed no effect of both propranolol and carvedilol on β-AR density in cultured cells. Dangel et al. [19] reported that in H9c2 cardiac cells, propranolol failed to increase receptor density after 24 h of treatment as determined by radioligand binding. Furthermore, Reynolds and Molinoff [20] observed no upregulation of β-ARs in S49 cells by propranolol after 24 h of treatment. Moreover, in cultured chick cardiac myocytes, Asano et al. [21] found that carvedilol itself did not downregulate β-AR density. Conversely, our results are in contrast to those of some previous studies. Flesch et al. [22] reported that carvedilol, at the concentration of 3 nM, decreased β-ARs in rat cardiomyocytes after 12 h of treatment. Furthermore, Hughes et al. [23] found that propranolol, at concentrations of 10–100 nM, downregulated β-ARs after 16–20 h of treatment of S49 or BC3H-1 cultured cells. Additionally, Reynolds and Molinoff [20] observed that pindolol, at the concentration of 20 nM, decreased total β-ARs in S49 cells after 24 h of treatment. At present, we are unable to explain the discrepancy between our results and those of other studies, although differences in cultured cells, drug concentrations, duration of the treatment, or techniques used for quantification of β-AR density may have contributed to the different results. Furthermore, since some β-AR antagonists have an elevated affinity for β-ARs and are considerably lipophilic, it is possible that the β-blocker is not completely removed during the experiment, thus inhibiting radioligand binding to the receptors by competition during incubation of biological samples with the radioligand and leading to an underestimation of the receptor density. In fact, it has been reported that, after carvedilol incubation of cells or human atrial trabeculae, the affinity of radioligands to β-ARs remained significantly reduced despite extensive efforts to remove carvedilol from receptors [22,24]. Whether other non-selective high-affinity β-blockers exhibit persistent binding to β-ARs even after washout in cell culture experiments remains to be ascertained. It is emphasized that in our study, receptor surface density was assayed by immunological methods, i.e., immunostaining followed by ELISA or by flow cytometry using a specific antibody that did not have such a disadvantage.

β-ARs are a family of G-protein coupled receptors comprising three subtypes, β_1_, β_2_, and β_3_, which act by activating a Gs protein and adenyl cyclase. β-AR signaling is terminated by phosphorylation of the intracellular domains of the receptor by the family of G protein-coupled receptor kinases. Chronic stimulation of β_2_-ARs by catecholamines causes desensitization of the β-adrenergic system with internalization and reduction of the number of receptors present on the cell surface. In the present study, we found that isoproterenol, a β-AR agonist, decreases β_2_-AR density without affecting β_2_-AR-mRNA levels in three different cells. We also found that β-blocker treatment leads to recycling receptors back to the surface, restoring receptor density, regardless of the ancillary pharmacological properties of β-blockers. These findings confirm and extend previous in vitro results showing the effects of catecholamines on receptor density and the impact of the treatment with the β-blocker propranolol on agonist-induced β_2_-AR downregulation. Di Certo et al. [14] showed that the exposure of cell surface β_2_-ARs to the β-AR agonist isoproterenol resulted in a time-dependent receptor downregulation. This effect was completely abolished by propranolol treatment.

The agonist-induced reduction of β_2_-AR cell surface density is a well-documented phenomenon. In contrast, little is known about the cell surface regulation of the β_1_-AR. Chronic stimulation of β-ARs by catecholamines causes internalization and reduction of the number of both β_1_- and β_2_-ARs present on the cell surface, with the former more resistant to agonist-mediated downregulation than the latter [7,8,9]. Previous experiments using engineered receptors and cultured cells have demonstrated that treatment with the β-blocker alprenolol is able to antagonize the actions of catecholamines and to restore β_1_-AR density [8]. However, at present, it is unknown whether alprenolol treatment itself is able to regulate β_1_-AR cell surface density in cultured cells.

Additional features of our experiments are worth commenting on. First, studies in humans and animals reported that long-term therapy with β-blockers alters receptor density. In the present study, the exposure of cells to β-AR antagonists lasted only a few hours. Although previous studies show that propranolol fails to increase receptor density after 24 h of treatment, it cannot be ruled out that very prolonged exposures (48–72 h) to β-blockers may influence the transcription and the number of β-ARs. Second, radioligand binding is a powerful and widely used method to quantitatively measure receptor surface density [25,26]. However, we also know from previous research that there are several technical and logistic challenges associated with this method [27]. Whole-cell ELISA provides a solid alternative to measure surface expression, and agonist-induced GPCR downregulation for N-terminally epitope-tagged receptors expressed in heterologous cells [28,29]. Third, to evaluate changes in endogenous β_2_-AR density, we used a flow cytometry-based method utilizing a specific antibody against β_2_-ARs according to previously reported studies [30,31]. Although flow cytometry is a rapid and reliable method for evaluating changes in receptor density, there are some caveats to consider. In particular, the method may require the optimization of temperature and incubation time to maximize the binding to the receptor, pre-incubation with human serum to decrease Fc binding, and the optimization of methods used for cell dissociation to avoid loss of cell surface epitopes. In addition, high-quality antibodies are needed. In our study, β-AR density changes were induced by very specific ligands. This greatly facilitated the optimization of flow cytometric analyses. Fourth, changes in lymphocyte β_2_-AR density can also be evaluated using the biotinylated β-AR ligand alprenolol [32]. However, it remains to be determined whether flow cytometric quantification of β_2_-AR density on blood cells, such as lymphocytes, may be useful to monitor treatment responses, disease activity, and prognosis of diseases involving changes in β-AR density, such as heart failure. Fifth, in the present study, we analyzed the effects of a subset of β-blockers with different signaling profiles on β_2_-AR density and found that their agonistic activity does not affect short-term regulation of β_2_-AR density. Since the three β-AR subtypes appear to differ in their distribution as well as their signaling properties, it remains to be determined whether the agonistic activity of β-blockers is able to regulate β_1_-AR and/or β_3_-AR cell surface density in cultured cells.

In conclusion, we evaluated the effects of a subset of β-blockers, endowed with varying degrees of G protein/β-arrestin efficacy profile, on β_2_-AR density and found that no tested β-blocker affected β_2_-AR density on their own but reversed agonist-induced β_2_-AR downregulation. Collectively these results suggest that the agonistic activity of β-blockers does not exert an important effect on short-term regulation of β_2_-AR density.

## 4. Materials and Methods

### 4.1. Materials

Isoproterenol, propranolol, pindolol, carvedilol, sotalol, timolol, anti-FLAG monoclonal antibody (M1 clone) and Hoechst 33,258 were from Sigma–Aldrich (St. Louis, MO, USA) while anti-β_2_ -AR antibody from Abcam (Cambridge, UK). Cell culture media, fetal bovine serum, G418, and Lipofectamine were from Invitrogen (Milan, Italy).

### 4.2. Cell Culture

Human embryonic kidney 293 (HEK 293) cells were grown in Dulbecco’s modified Eagle’s medium (DMEM, GIBCO, Life Technologies, Monza, Italy) supplemented with 10% fetal bovine serum (FBS, GIBCO, Life Technologies, Monza, Italy), 100 units/mL penicillin, 100 μg/mL streptomycin sulfate, and 200 μg/mL G418 (GIBCO, Life Technologies, Monza, Italy) in a humidified atmosphere of 5% CO2 at 37 °C. Cultured cells were serum-starved for 2 h prior to agonist stimulation.

HL-1 cardiac muscle (HL-1) cells were kindly provided by Dr. S. Nanni (Università Cattolica del Sacro Cuore, Rome, Italy) and were cultured in Claycomb medium (Sigma–Aldrich, St. Louis, MO, USA) supplemented with 10% FBS, 0.2 mM norepinephrine, 2 mM L-glutamine, 1 U/mL penicillin, and 1 μg/mL streptomycin solution (Sigma–Aldrich, St. Louis, MO, USA) as previously described [33].

### 4.3. Isolation of Human Lymphocytes

Three healthy donors (38 ± 3.6 years of age) were recruited for this study at the Institute of Hematology, Sapienza University of Rome (Italy). Following the rules of good medical practice, the nature and purpose of the study were explained to the volunteers who then gave their informed consent. The investigation conforms to the principles outlined in the Declaration of Helsinki. Lymphocytes were isolated from the peripheral blood by centrifugation on a Ficoll/Hypaque density gradient and plastic adherence to deplete monocytes before pharmacological treatments.

### 4.4. Fluorescence Microscopy

After pharmacological treatments, HL-1 cells were washed in PBS and then stained in ice with an anti-β_2_-AR antibody (1:100, ab61778, Abcam, Cambridge, UK) for 45 min, following the manufacturer’s instructions. The anti-β_2_-AR antibody was chosen based on previously reported data [30,31]. After washing, cells were incubated with AlexaFluor 488-conjugated anti-rabbit IgG (Invitrogen Corporation, Milan, Italy) as a secondary antibody for an additional 30 min. At the end of staining, cells were fixed in 2% paraformaldehyde for 15 min, counterstained with Hoechst 33,258 (Sigma–Aldrich, St. Louis, MO, USA) at the concentration of 1 mg/mL in PBS, and then mounted in glycerol/PBS (ratio 1:1, pH 7.4). Images were acquired by intensified video microscopy (IVM) with an Olympus fluorescence microscope (Olympus Corporation, Milan, Italy) equipped with a Zeiss charge-coupled device (CCD) camera (Carl Zeiss, Oberkochen, Germany).

### 4.5. Flow Cytometry

The effects of β_2_-AR agonists and antagonists on receptor density were also analyzed using flow cytometry. At the end of pharmacological treatments, human lymphocytes and cardiac HL-1 cells were fixed with 0.01% paraformaldehyde for 10 min to avoid changes in cell surface receptors. After washing in PBS, cells were stained with anti-β_2_-AR (1:100, ab61778, Abcam, Cambridge, UK) for 45 min, following the manufacturer’s instructions. As mentioned above, the anti-β_2_-AR antibody was chosen based on previously reported data [29,30]. Next, cells were incubated with AlexaFluor 488-conjugated secondary antibody (Invitrogen Corporation, Milan, Italy) for an additional 30 min. Isotypic immunoglobulines followed by secondary antibody were used as a negative control (rabbit IgG, polyclonal–ab37415, Abcam, Cambridge, UK). Stained cells were analyzed using a FACSCalibur flow cytometer (BD Biosciences, San Jose, CA, USA) equipped with a 488 argon laser and with a 635 red diode laser. At least 30,000 events per sample were acquired and analyzed using the Cell Quest Pro software (BD Biosciences, San Jose, CA, USA). The median values of fluorescence intensity were used to provide a semi-quantitative evaluation. Changes in receptor density levels were determined as a difference in the fluorescence intensity in control and treated cells.

### 4.6. Receptor Downregulation and Recycling Assays

HEK293 cells stably expressing the N-terminus-FLAG-tagged β_2_-AR were incubated in the presence of vehicle (control), propranolol, carvedilol, pindolol, sotalol, timolol, or isoproterenol at the concentration of 1 μM at 37 °C per 3 h. Concentrations of β_2_-AR ligands were chosen based on results from previous studies [34,35]. Isoproterenol-induced receptor downregulation was reversed by adding 1 μM of the β-blocker to the cells, which were then incubated at 37 °C for an additional hour. The reaction was stopped by fixing the cells with 2% paraformaldehyde in phosphate buffer saline (PBS). Then, the cells were subjected to immune-stained ELISA to quantify the surface receptor density. Briefly, without permeabilization, cells were blocked in 5% nonfat-dried milk in PBS and stained with anti-FLAG-M2-alkaline phosphatase-conjugated antibody (Sigma–Aldrich, St. Louis, MO, USA) at the concentration of 0.1 mg/mL for 1 h at room temperature. The unbound antibody was removed by washing four times with PBS. Next, alkaline phosphatase substrate was added to the cells, and luminescence was immediately read using a plate luminometer (Victorlight, Perkin Elmer, Milan, Italy).

### 4.7. RNA Isolation and Quantification

Total RNA was extracted from cultured cells and ventricular tissues by using TRIzol (Invitrogen, Monza, Italy) and purified by using an RNA purelink mini kit (Invitrogen, Monza, Italy). The concentration and purity of the RNA solution were determined by using a NanoDrop spectrophotometer (Fisher Scientific, Monza, Italy), whereas its overall quality was analyzed using the Agilent 2100 bioanalyzer with an RNA LabChip (RNA 6000 Nano kit, Agilent, Milan, Italy). cDNA was obtained using the High Capacity cDNA Archive kit (Applied Biosystems, Foster City, CA, USA). mRNA expression levels of the β_2_-AR were performed using TaqMan gene expression assays (code n. Mm02524224_s1 and Hs00240532_s1 for HL-1 cells and lymphocytes, respectively) (Applied Biosystems, Foster City, CA, USA). qRT-PCR analysis was performed using the 7500 Real-Time PCR system (Applied Biosystems, Foster City, CA, USA). The glyceraldehyde 3-phosphate dehydrogenase (GAPDH, code n Mm99999915_g1 and Hs02786624_g1 for HL-1 cells and lymphocytes, respectively) gene was used as a reference gene, and the ΔCt was used for statistical analysis.

### 4.8. Statistical Analysis

Data are expressed as the mean ± SEM and analyzed using a software program (GraphPad Prism version 5.03, GraphPad Software Inc., San Diego, CA, USA). Statistical significance between different groups was determined by unpaired *t*-test for two groups or one-way ANOVA with Bonferroni’s post hoc test to compare all pairs of columns for more than two groups. A value of *p* < 0.05 was considered statistically significant.

## 5. Conclusions

In the present study, β-blockers endowed with inverse, partial, or biased agonism at the β_2_-AR were evaluated for their effects on β_2_-AR density in both cells expressing exogenous FLAG-tagged human β_2_-ARs and cells expressing endogenous β_2_-ARs. We found that β-blockers do not affect β_2_-AR density on their own but reverse isoproterenol-induced β_2_-AR downregulation. These results suggest that the agonistic activity of β-blockers does not exert an important effect on short-term regulation of β_2_-AR density.
